# Oxidative Stress, Antioxidant Capacity, Dyslipidemia and Cardiovascular Risk in Sickle Cell Disease: A Systematic Review and Meta‐Analysis

**DOI:** 10.1155/tswj/8864775

**Published:** 2026-03-12

**Authors:** Josué Louokdom Simo, Romaric De Manfouo Tuono, Maryline Seuko Njopwouo, Claude Tagny Tayou

**Affiliations:** ^1^ Department of Medicine and Biomedical Sciences, Higher Institute of Health Sciences of Université des Montagnes, Bangangté, Cameroon; ^2^ Department of Medicine of the Faculty of Medicine and Biomedical Sciences, Université de Yaoundé 1, Yaoundé, Cameroon, uninet.cm

**Keywords:** cardiovascular risk, dyslipidemia, oxidative stress, sickle cell disease

## Abstract

**Background:**

Sickle cell disease is a human hemoglobinopathy associated with high hemolytic capacity. Hemoglobin S (HbS) polymerization is a primary pathophysiological event in sickle cell anemia. Despite numerous studies conducted to understand the pathophysiology of the disease, including oxidative imbalance and cardiovascular risk, questions still arise. This systematic review and meta‐analysis aimed to evaluate studies linking oxidative stress, lipid profile, and dyslipidemia in sickle cell patients, predisposing them to atherogenic risk.

**Methods:**

The systematic review of databases and search engines was conducted over 24 years (2000–2024) worldwide, according to the guidelines of PRISMA and the Cochrane Handbook. Research articles were searched in the PubMed and Web of Science databases. Only case‐control articles were retained. Data were extracted from the articles and analyzed using R version 4.3.2, with a common‐effect model for meta‐analyses. Heterogeneity was assessed using *I* ^2^ statistics. The standardized mean difference (SMD) was used to assess the extent of the disease on the different parameters studied. Heterogeneity across individual studies was assessed using Higgins’s inconsistency Q statistics and reported as *I* ^2^ and *p*‐values. ROBINS‐E was used to assess the risk of bias in the included studies.

**Results:**

A total of 405 studies were initially identified; after the elimination of duplicates and studies not meeting the objectives of the review, 25 studies were finally included in the meta‐analyses. The reported SMDs using a common‐effect model were 1.15 [0.96; 1.34] (*p* <0.01) for Lactate Dehydrogenase (LDH), 1.03 [0.48; 1.53] (*p* <0.01) for Myeloperoxidase (MPO), and 1.13 [0.96; 1.29] (*p* <0.01) for Malondialdehyde (MDA), reflecting the strong influence of sickle cell disease on hemolysis and the production of lipid peroxidation markers compared with normal controls. The antioxidant markers Glutathione Peroxidase (GPx), Reduced Glutathione (GSH), catalase, Superoxide Dismutase (SOD), and Total Antioxidant Capacity (TAC) reported respective SMDs of −1.97 [−2.32; −1.63] (*p* <0.01), −3.01 [−3.50; −2.52] (*p* <0.01), −1.39 [−1.58; −1.20] (*p* <0.01), −1.99 [−1.92; −1.47] (*p* <0.01), and−1.58 [−1.90; −1.25] (*p* <0.01), reflecting the strong negative influence of sickle cell disease on the activity of these enzymes. The evaluated lipid profiles reported dyslipidemia and an atherogenic risk characterized by a significant negative influence on plasma concentrations of Total Cholesterol (TC), High‐Density Lipoprotein cholesterol (HDLc), and Low‐Density Lipoprotein cholesterol (LDLc), with respective SMDs of −1.32 [−1.42; −1.21] (*p* <0.01), −0.84 [−0.94; −0.73] (*p* <0.01), and−2.54 [−3.15; −1.93]. Conversely, a significant influence of sickle cell disease was observed on the triglycerides/HDL‐c ratio and triglycerides, characterized by respective SMDs of 1.58 [1.41; 1.75] (*p* <0.01) and 1.58 [1.41; 1.75] (*p* <0.01).

**Conclusion:**

Analyses performed in these studies reported a large influence of sickle cell disease on oxidative stress, characterized by an imbalance of the oxidant/antioxidant system in favor of oxidants, as well as lipid profile imbalance causing dyslipidemia and a high atherogenic risk compared with normal controls, as assessed by large SMDs. These results provide additional information on oxidative stress abnormalities in sickle cell disease and may serve as a basis for decision‐making aimed at improving patient care.

## 1. Introduction

Sickle Cell Disease (SCD) is a human hemoglobinopathy associated with a high hemolytic power, resulting from the formation of HbS due to a mutation in the *β*‐globin chain, in which glutamic acid at position 6 is replaced by valine [1, 2]. HbS polymerization is a primary pathophysiological event in sickle cell anemia [3]. The phenotypic expression of sickle cell disease is a complex pathophysiological condition that exposes patients to multiple sources of pro‐oxidants, leading to chronic consequences and systemic oxidative stress [4, 5]. Indeed, due to its abnormal structure, the sickle red blood cell is frequently subjected to factors that increase the intraerythrocyte concentration of HbS and consequently polymerization, including dehydration, metabolic acidosis, and cellular magnesium depletion [6, 7].

In a state of deoxygenation (a decrease in the partial pressure of oxygen [PaO2]), the higher the intraerythrocyte concentration of HbS, the lower the solubility of the polymers; this results in polymerization kinetics proportional to the concentration of HbS within the cell [8, 9]. At the cellular level, this polymerization leads to the formation of helical fibers, causing deformation, stiffening, and weakening of cells, and consequently sickling and fragmentation of red blood cells [10, 11]. It also induces intraerythrocyte dehydration concomitant with increased membrane permeability due to abnormalities in ion transport. This dehydration, generated by polymerization, further increases the intraerythrocyte concentration of HbS and its insolubility, thereby establishing a vicious cycle [12–14].

Simultaneously, polymerization alters the phospholipid architecture of the red blood cell membrane. This instability is promoted by the presence of an oxidizing microenvironment secondary to the release of ferric iron (Fe^3+^) following HbS denaturation [10, 11, 15]. In fact, the resulting oxidative stress corresponds to the activation of molecular oxygen, leading to a cascade of radical reactions [16, 17]. These reactions generate free radicals (atoms or molecules carrying an unpaired electron) characterized by a very short lifespan and extremely high chemical reactivity. Hyperreactive oxygen species are mainly represented by the superoxide anion (O2^-^), the highly reactive and non‐diffusible hydroxyl radical (OH·), as well as singlet oxygen [4, 18].

Under these conditions, oxygen becomes responsible for highly toxic chemical reactions within cells, particularly when the body’s natural antioxidant defenses are deficient or absent, such as deficiencies in vitamins A and E, or insufficiency of enzymatic barriers including superoxide dismutase, catalase, and glutathione reductase. Among the biochemical lesions, membrane lipid peroxidation (LPO) is the most extensively described and is evidenced by the production of oxidative stress markers, including malondialdehyde (MDA) [19, 20].

In sickle cell disease, the mechanisms of free radical production are explained by various processes corresponding to the pleiotropic effects of the mutant gene [21, 22]. Furthermore, low‐density lipoprotein (LDL) can be oxidized through radical mechanisms involving trace metals, which play an important role in the genesis of atherosclerosis. LDL represents the main form of cholesterol transport to tissues and thus constitutes a major factor in atherogenic risk [23, 24]. Altogether, these findings clearly demonstrate that, among the various aspects of the pathophysiology, free radical production and subsequent lipid peroxidation are major events contributing to the reduction in red blood cell lifespan and the development of anemia [25, 26]. Finally, in sickle cell disease, disturbances in oxidative stress, lipid metabolism, and serum lipoproteins constitute consecutive factors accelerating the arteriosclerotic process in affected patients [27].

The pathophysiology of sickle cell disease is complex, and isolated studies have explored specific aspects of this condition. The objective of this study is to review the literature and conduct a meta‐analysis in order to highlight the imbalance of oxidative stress in sickle cell patients in favor of pro‐oxidants, and to present these abnormalities as contributing factors to dyslipidemia and, consequently, cardiovascular risk. This study could serve as a basis for information and decision‐making aimed at improving the management of sickle cell patients.

## 2. Methodology

This study is a systematic review and meta‐analysis addressing the central question of whether inflammatory and hemostatic abnormalities in patients with sickle cell disease predispose them to an increased risk of thrombosis. The systematic review was conducted in accordance with the Preferred Reporting Items for Systematic Reviews and Meta‐Analyses (PRISMA) guidelines [28] and the Cochrane Handbook for Systematic Reviews of Interventions [29], which guided the database and search engine screening process.

### 2.1. Search Strategy and Study Selection

The search was conducted in the following databases: MEDLINE/PubMed and Web of Science. These databases were selected to ensure the rigor and quality of article selection from indexed journals. The search strategy was constructed using MeSH terms by combining the following main keywords: *“Sickle Cell Disease + Dyslipidemia + Cardiovascular Risk”*, *“Sickle Cell Disease + Oxidative Stress + Dyslipidemia”*, and *“Sickle Cell Disease + Oxidative Stress + Dyslipidemia + Cardiovascular Risk”*. The search period covered studies published between 2000 and 2024. Studies that met the objectives of the review were included, while those that did not were excluded. In addition, only published studies were considered; grey literature and unpublished studies were not included.

### 2.2. Study Selection and Data Extraction

To better address the research question of this study, only case–control studies were included. Studies of other design were not retained. Only studies published in English were considered. To ensure an exhaustive selection of research conducted during the study period, the predefined search strategy and protocol were applied by two experienced researchers. The titles and abstracts of all publications retrieved through the search strategy were independently reviewed by two reviewers. The selection and identification of studies to be included were conducted by mutual agreement. The same reviewers independently assessed the full texts of the selected studies for eligibility. Consequently, articles that met the selection criteria were retained, and after further evaluation, they were finally validated for definitive inclusion and eligibility for the data extraction phase. Any disagreements were resolved by consensus. All included articles were assessed using the ROBINS‐E tool, which is designed to evaluate the risk of bias in non‐randomized studies of exposure [30]**.**

Data extraction from the selected articles was performed using a Microsoft Excel spreadsheet (version 2019). A second review was conducted by another experienced researcher to verify the accuracy and consistency of the extracted data. The information extracted from the articles mainly included the following:

• **Study information:** type of article, year of publication, author name, country and continent of publication, and journal name;
• **Study setting and sample characteristics:** sample size, sex, and age of the study population;
• **Methods used:** study design and types of interventions;
• Main results obtained.

### 2.3. Risk of Bias Assessment

**Figure 1:**
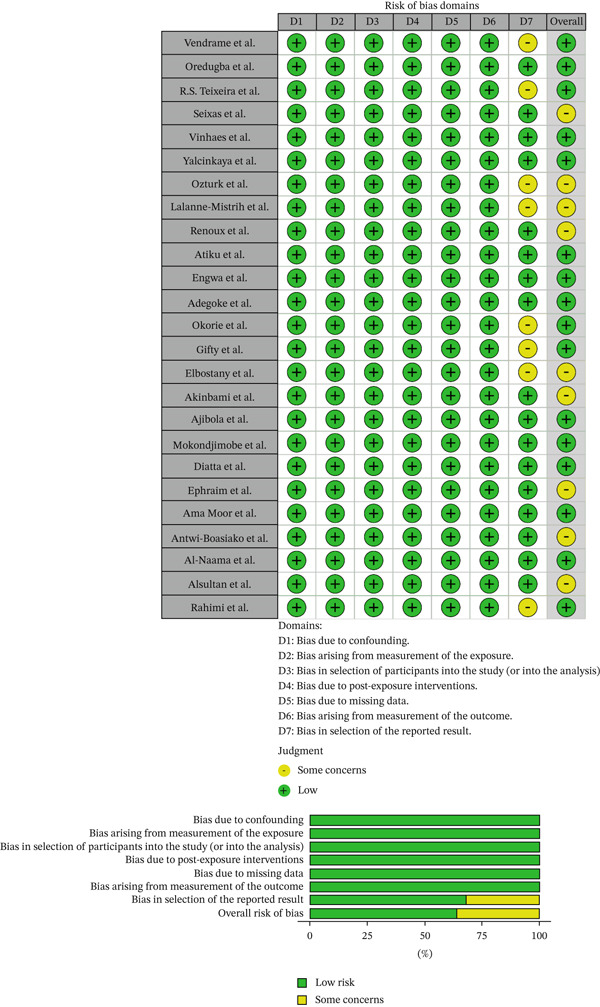
Assessment of risk of bias of non‐randomized studies of exposure effects using ROBINS‐E [30].

ROBINS‐E, a tool for assessing the risk of bias in non‐randomized studies of exposure effects [30]**,** was used to assess the risk of bias in the included studies. The ROBINS‐E risk‐of‐bias assessment covers seven domains of bias, as presented in Figure 1. Each domain is addressed using a series of signaling questions aimed at eliciting relevant information about the study and the analysis being assessed. Most questions have the response options “Yes”, “Probably yes”, “Probably no”, “No”, and “No information”. Responses of “Yes” and “Probably yes” have the same implications for risk of bias, as do “No” and “Probably no” [30].

The bias domains analyzed were: bias due to confounding; bias arising from measurement of the exposure; bias in the selection of participants into the study (or into the analysis); bias due to post‐exposure interventions; bias due to missing data; bias arising from measurement of the outcome; and bias in the selection of the reported result. These data were entered into an Excel 2016 file and analyzed using the aforementioned tool.

### 2.4. Data Synthesis, Management, Study Quality Assessment, and Analysis

The statistical software R version 5 (version 4.3.2, The R Foundation for Statistical Computing, Vienna, Austria) was used to analyze and graphically present the results. A narrative description, including a presentation of the included studies, was provided by country, continent, and worldwide. Thus, the standardized mean difference (SMD) parameter was used to determine the mean score and the effect size between the intervention and control groups and to assess differences, as defined by Wan et al. (2014) in their study entitled *“Estimating the sample mean and standard deviation from the sample size, median, range and/or interquartile range”* [31]. The SMD was interpreted as follows: a weak effect for values between 0.2 and<0.3, a moderate effect for values between 0.3 and<0.8, and a large effect for values >0.8. A negative value indicates that the experimental group is associated with a decrease in the evaluated parameter [32]. For this study, the SMD was reported using the common‐effect model, as the included studies were almost identical in terms of protocols and populations. Heterogeneity across individual studies was assessed using Higgins’s inconsistency Q statistic and reported as I^2^ and *p*‐values. Heterogeneity among included studies was interpreted as follows: low heterogeneity for *I* ^2^ values <0.25 (25%), moderate heterogeneity for values between 0.25 (25%) and 0.5 (50%), and significant heterogeneity for values >0.5 (50%) [33]. For the different comparisons performed, a *p*‐value <0.05 was considered statistically significant, with a confidence interval of 95%.

## 3. Results

### 3.1. Revision Process

**Figure 2:**
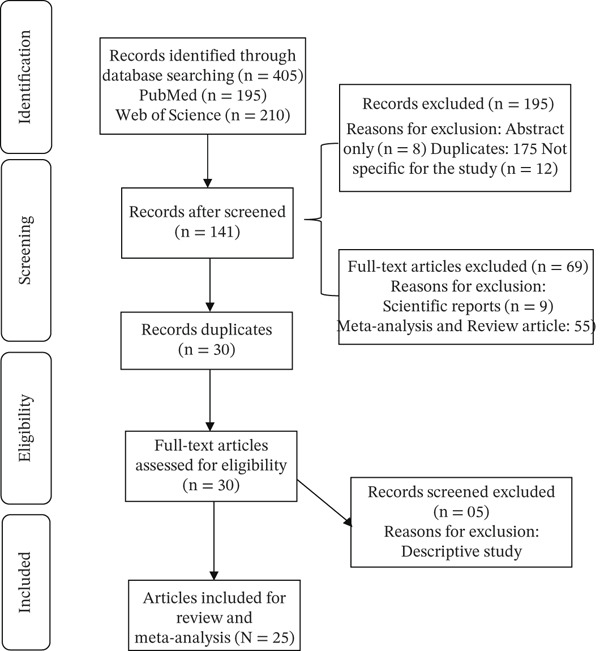
Flow diagram of the literature search.

Initially, after entering the keywords into the various databases, 405 search results were obtained (195 from PubMed and 210 from Web of Science). After eliminating journal‐type articles, meta‐analyses, articles whose content was not accessible (only the abstract), and those that did not address the research question, 141 articles were retained for more in‐depth analysis. Of these, after removing duplicates from the databases used, 30 articles were retained. Of the 405 initially identified studies, 25 were ultimately included after screening for duplicates, accessibility, and relevance (Figure 2: flow chart describing the article selection process).

### 3.2. Characteristics and Description of the Included Studies

The Table 1 presents the basic characteristics of the 25 included studies.

**Table 1:** Basic characteristics of the included literature. First.

First author	Published year	Experimental group	Control group	Country	Continent
Vendrame	2019	40	30	Usa	America
Oredugba	2002	40	37	Usa	America
R.S. Teixeira	2019	55	41	Brazil	S‐America
Seixas	2010	152	132	Brazil	S‐America
Vinhaes	2020	40	27	Brazil	S‐America
Yalcinkaya	2019	35	19	Türkiye	Europe
Ozturk	2013	45	38	Türkiye	Europe
Lalanne‐Mistrih	2018	97	1010	France	Europe
Renoux	2017	62	12	France	Europe
Atiku	2019	51	51	Uganda	Africa
Engwa	2021	40	36	Nigeria	Africa
Adegoke	2016	62	40	Nigeria	Africa
Okorie	2018	30	22	Nigeria	Africa
Gifty	2020	50	34	Ghana	Africa
Elbostany	2023	90	45	Egypt	Africa
Akinbami	2019	152	152	Nigeria	Africa
Ajibola	2019	32	50	Nigeria	Africa
Mokondjimobe	2012	41	48	Congo	Africa
Diatta	2014	43	42	Senegal	Africa
Ephraim	2016	50	50	Ghana	Africa
Ama Moor	2016	42	42	Cameroon	Africa
Antwi‐Boasiako	2019	34	50	Ghana	Africa
Al‐Naama	2015	42	50	Iraq	Asia
Alsultan	2010	51	50	Saudi Arabia	Asia
Rahimi	2006	62	24	Iran	Asia

The included studies have the following basic characteristics: 5 articles from America with the following authors: Vendrame et al. (2019) [34], Oredugba et al. (2002) [35], Vinhaes et al. (2020) [36], Seixas et al. (2010) [37], Machado et *al*. (2019) [38]; 4 articles from Europe including the following authors Yalcinkaya et al. (2019) [39], Ozturk et al. (2013) [40], Lalanne‐Mistrih et al. (2018), Renoux et *al*. (2018) [41]; 13 studies from Africa by the following authors: Atiku et al. (2019) [19], Engwa et al. (2021) [42], Adegoke et al. (2016) [43], Okorie et al. (2018) [44], Gifty et al. (2020) [45], Elbostany et al. (2023) [20], Akinbami et al. (2019) [23], Ajibola et al. (2019) [26], Mokondjimobe et al. (2012) [46], Diatta et al. (2014) [47], Ephraim et al. (2016) [24], Ama Moor et *al*. (2016) [48], Antwi‐Boasiako et *al*. (2019) [49]; 3 studies from Asia by the following authors: Al‐Naama et al. (2015) [50], Alsultan et *al*. (2010) [51] et Rahimi et *al*. (2006) [52].

### 3.3. Results of Risk of Bias Assessment

Figure 1 below shows the results obtained from the risk of bias assessment using the ROBINS‐E tool:

After assessment, although some studies presented “some concerns,” all included studies generally reported a “low” risk of bias (as shown in Figure 1), with a heterogeneity (*I* ^2^) of approximately 62.5%, thus generally supporting and justifying the presence of statistically significant differences between individuals with sickle cell disease (HbSS) and controls (HbAA).

### 3.4. Sickle Cell Disease and Oxidative Stress Outcomes of the Included Articles

#### 3.4.1. Hemolysis Marker: LDH From Included Studies Outcomes of the Included Articles

**Figure 3:**
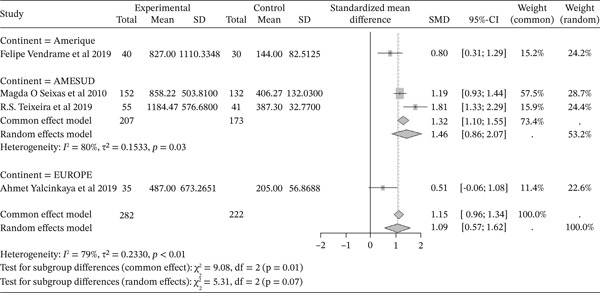
Representation of LDH from included studies. I^2^ = heterogeneity index; *τ* ^2^ = between‐study variance; SD = Standard; SMD = standardized mean difference; CI 95% = confidence interval (CI) at 95%.

Figure 3 shows the representation of LDH activity from the included studies. Of these studies, four explored LDH in cases and controls, including one in America, two in South America, and one in Europe. The total population size across these studies was 504, including 282 cases and 222 controls. The reported SMD using a common‐effect model for these studies is 1.15 [0.96; 1.34] (p <0.01), reflecting the strong influence of sickle cell disease on hemolysis compared to normal controls. The study reporting the greatest influence is that of Teixeira et al. (2019) [38] from the American continent, with an SMD of 1.88 [1.33; 2.29], while the study reporting the lowest influence was conducted in the United States by Vendrame et al. (2019) [34] with an SMD of 0.8 [0.31; 1.29]. It should be noted that heterogeneity was observed (*I* ^2^>50%).

#### 3.4.2. MPO Activity From Included Studies, Outcomes of the Included Articles

**Figure 4:**
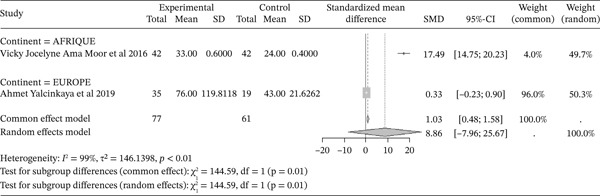
Representation of MPO from included studies. I^2^ = heterogeneity index; *τ* ^2^ = between‐study variance; SD = Standard; SMD = standardized mean difference; CI 95% = confidence interval (CI) at 95%.

Figure 4 shows the representation of MPO activity from the included studies. Of these studies, two evaluated the influence of sickle cell disease on MPO activity compared to controls: one in Africa by Ama Moor et al. (2016) [48] and one in Europe by Yalcinkaya et al. (2019) [39]. The total population size across these studies was 138, including 77 cases and 61 controls. The reported SMD using a common‐effect model for these studies is 1.03 [0.48; 1.53] (p <0.01), reflecting the strong influence of sickle cell disease on MPO activity compared to normal controls. The study by Ama Moor et al. (2016) reported the strongest effect [48]. It should be noted that heterogeneity was observed (I^2^>50%).

#### 3.4.3. Membrane Lipid Peroxidation Products: MDA From the Included Studies’ Outcomes of the Included Articles

**Figure 5:**
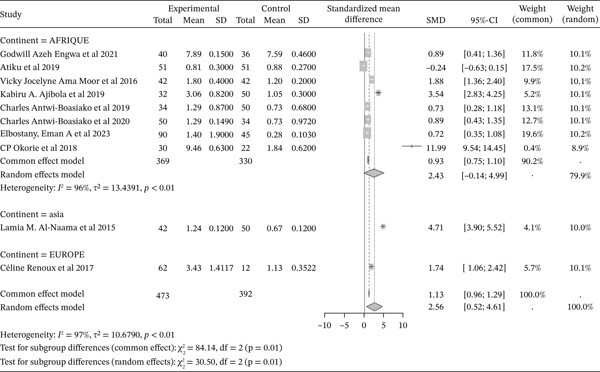
Representation of MDA from included studies. Legend: I^2^ = heterogeneity index; *τ* ^2^ = between‐study variance; SD = Standard; SMD = standardized mean difference; CI 95% = confidence interval (CI) at 95%.

Figure 5 shows the representation of MDA concentration from the included studies. Of these studies, ten investigated the influence of sickle cell disease on membrane lipid peroxidation products (MDA), including eight in Africa, one in Asia, and one in Europe. The total population across these studies was 865, including 473 cases and 392 controls. The reported SMD using a common‐effect model is 1.13 [0.96; 1.29] (p <0.01), reflecting a strong influence of sickle cell disease on membrane lipid peroxidation measured by MDA. The study reporting the highest SMD was Okorie et al. (2018) from Africa [44], with a SMD of 11.99 [9.54; 14.45], while the study with the lowest effect was Atiku et al. (2019) [19]. The included studies showed significant heterogeneity (*I* ^2^>50%).

#### 3.4.4. Gpx Activity From the Included Studies Outcomes of the Included Articles

**Figure 6:**
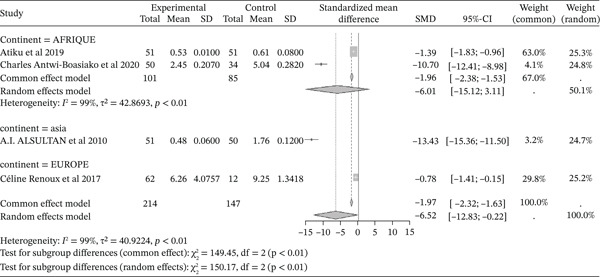
Representation of GPx from included studies. Legend: I^2^ = heterogeneity index; *τ* ^2^ = between‐study variance; SD = Standard; SMD = standardized mean difference; CI 95% = confidence interval (CI) at 95%.

Figure 6 shows the representation of GPx activity from the included studies. Four of the included studies investigated the influence of sickle cell disease on GPx activity, with a total combined population of 361, including 214 cases and 147 controls. The reported SMD using a common‐effect model is ‐1.97 [‐2.32; ‐1.63] (p <0.01), reflecting a strong negative influence of sickle cell disease on GPx activity. The study reporting the strongest effect is that of Alsultan et al. (2010) from Asia [51], with an SMD of ‐13.43 [‐13.36; ‐11.50]. Significant heterogeneity was observed across the included studies (I^2^>50%).

#### 3.4.5. Concentration of GSH From Included Studies Outcomes of the Included Articles

**Figure 7:**
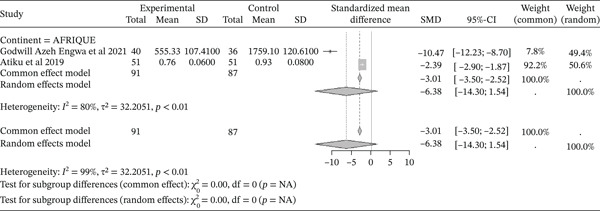
Representation of GSH from included studies. Legend: I^2^ = heterogeneity index; *τ* ^2^ = between‐study variance; SD = Standard; SMD = standardized mean difference; CI 95% = confidence interval (CI) at 95%.

Figure 7 shows the representation of GSH concentration from the included studies. Two of the included studies evaluated the influence of sickle cell disease on GSH production, both conducted in Africa. The total population size of these studies is 178, including 91 cases and 87 controls. The reported SMD using a common‐effect model is ‐3.01 [‐3.50; ‐2.52] (p <0.01), reflecting a strong negative influence of sickle cell disease on GSH activity. The study reporting the strongest effect is that of Engwa et al. (2021), with an SMD of ‐10.47 [‐12.23; ‐8.70] [42]. Significant heterogeneity was observed across the included studies (I^2^>50%).

#### 3.4.6. Activity of Catalase From Included Studies Outcomes of the Included Articles

**Figure 8:**
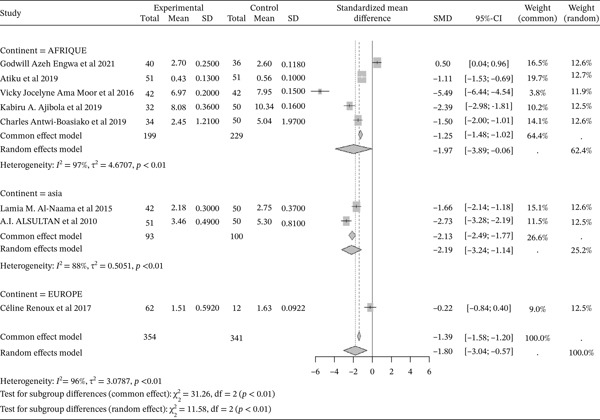
Representation of catalase activity from included studies. Legend: I^2^ = heterogeneity index; *τ* ^2^ = between‐study variance; SD = Standard; SMD = standardized mean difference; CI 95% = confidence interval (CI) at 95%.

Figure 8 shows the representation of catalase activity from the included studies. Eight of the included studies evaluated the influence of sickle cell disease on catalase activity compared to controls, including five from Africa, two from Asia, and one from Europe. The total population for the combined studies is 695, including 354 cases and 341 controls. The reported SMD using a common‐effect model is ‐1.39 [‐1.58; ‐1.20] (p <0.01), reflecting the negative impact of sickle cell disease on catalase activity. The study reporting the strongest effect is that of Ama Moor et al. (2016) from Africa [48], while the study showing the lowest effect is that of Engwa et al. (2021) [42]. Significant heterogeneity was observed across the included studies (I^2^>50%).

#### 3.4.7. Activity of SOD From Included Studies Outcomes of the Included Articles

**Figure 9:**
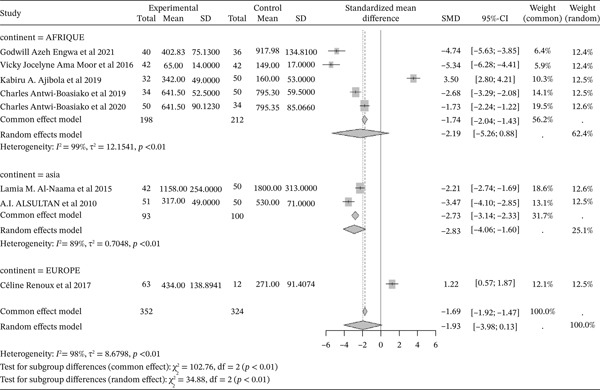
Representation of SOD from included studies. Legend: I^2^ = heterogeneity index; *τ* ^2^ = between‐study variance; SD = Standard; SMD = standardized mean difference; CI 95% = confidence interval (CI) at 95%.

Figure 9 shows the representation of SOD activity from the included studies. Eight of the included studies evaluated the influence of sickle cell disease on SOD activity compared to controls, including five from Africa, two from Asia, and one from Europe. The total population for the combined studies is 677, including 353 cases and 324 controls. The reported SMD using a common‐effect model is ‐1.99 [‐1.92; ‐1.47] (p <0.01), reflecting the negative impact of sickle cell disease on SOD activity. The study reporting the strongest effect is that of Engwa et al. (2021) from Africa [42], while the study showing the weakest effect is that of Ajibola et al. (2019) from Africa [26]. Significant heterogeneity was observed across the included studies (I^2^>50%).

#### 3.4.8. Representation of **TAC** From Included Studies Outcomes of the Included Articles

**Figure 10:**
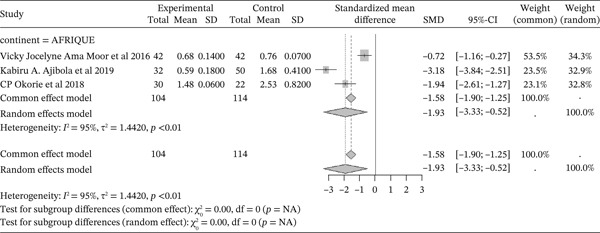
Representation of TAC from included studies. Legend: I^2^ = heterogeneity index; *τ* ^2^ = between‐study variance; SD = Standard; SMD = standardized mean difference; CI 95% = confidence interval (CI) at 95%.

Figure 10 shows the representation of Total Antioxidant Capacity (TAC) from the included studies. Three of the included studies, all from Africa, evaluated the influence of sickle cell disease on TAC. The total population for the combined studies is 218, including 104 cases and 114 controls. All studies reported a significant negative effect of sickle cell disease on this marker, with a reported SMD using a common‐effect model of ‐1.58 [‐1.90; ‐1.25] (p <0.01). The study reporting the strongest negative effect is that of Ajibola et al. (2019) [26]. Significant heterogeneity was observed across the included studies (I^2^>50%).

### 3.5. Sickle Cell Disease and Lipid Profile

#### 3.5.1. Sickle Cell Disease and Total Cholesterol Outcomes of the Included Articles

**Figure 11:**
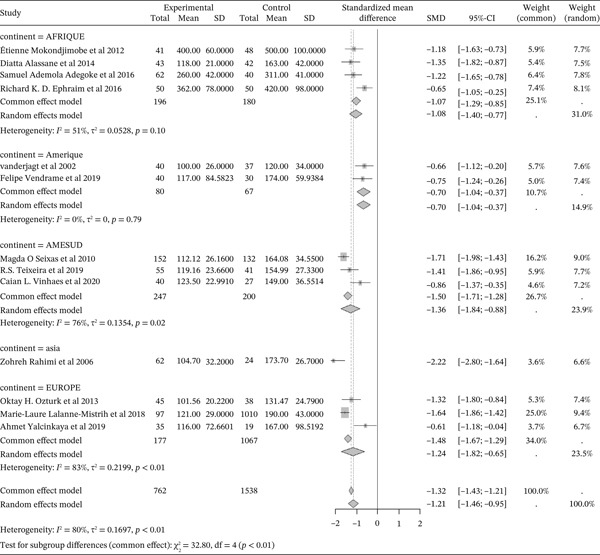
Representation of total Cholesterol from included studies. Legend: I^2^ = heterogeneity index; *τ* ^2^ = between‐study variance; SD = Standard; SMD = standardized mean difference; CI 95% = confidence interval (CI) at 95%.

Figure 11 shows the representation of Total Cholesterol from the included studies. Thirteen of the included studies evaluated the influence of sickle cell disease on plasma total cholesterol concentrations, including 4 from Africa, 2 from America, 3 from South America, 1 from Asia, and 3 from Europe. The total population size for these studies is 2,300, including 762 cases and 1,538 controls. The results indicate a significant negative influence of sickle cell disease on plasma total cholesterol concentrations, with a reported SMD using a common‐effect model of ‐1.32 [‐1.42; ‐1.21] (p <0.01). The study reporting the strongest influence is that of Rahimi et al. (2006) from Asia, with an SMD of ‐2.22 [‐2.80; ‐1.64,] [52]. Conversely, the study showing the weakest influence is that of Yalcinkaya et al. (2019) from Europe [39], with an SMD of ‐0.61 [‐1.18; ‐0.04]. Significant heterogeneity was observed across the included studies (I^2^>50%).

#### 3.5.2. Sickle Cell Disease and HDL Cholesterol Outcomes of the Included Articles

**Figure 12:**
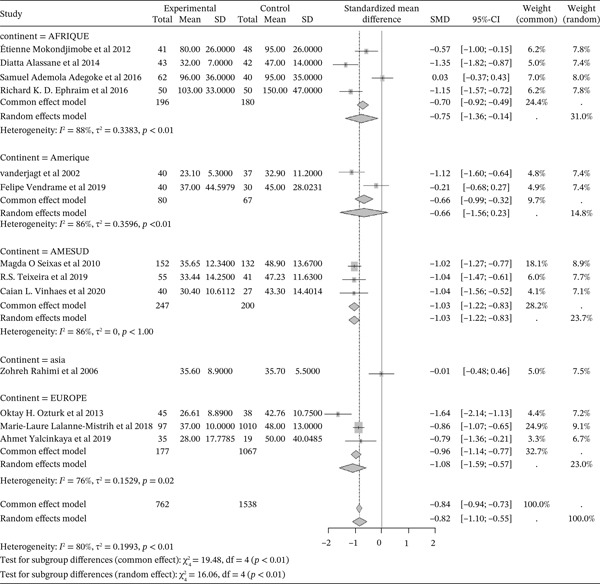
Representation of HDL Cholesterol from included studies. Legend: I^2^ = degree of heterogeneity, ??^2^ = degree of bias; SD = Standard Deviation; SMD = Standardized Mean Difference; CI 95% = confidence interval (CI) at 95%.

Figure 12 shows the representation of HDL Cholesterol from the included studies. Thirteen of the included studies evaluated the influence of sickle cell disease on plasma HDL cholesterol concentrations, including 4 from Africa, 2 from America, 3 from South America, 1 from Asia, and 3 from Europe. The total population size for these studies is 2,300, including 762 cases and 1,538 controls. The results indicate a significant negative influence of sickle cell disease on plasma HDL cholesterol concentrations, with a reported SMD using a common‐effect model of ‐0.84 [‐0.94; ‐0.73] (p <0.01). The study reporting the strongest influence is that of Ozturk et al. (2013) from Europe, with an SMD of ‐1.64 [‐2.14; ‐1.13] [40]. Conversely, the study showing the weakest influence is that of Adegoke et al. (2016) from Africa, with an SMD of ‐0.61 [‐1.18; ‐0.04] [43]. Significant heterogeneity was observed across the included studies (I^2^>50%).

#### 3.5.3. Sickle Cell Disease and LDL Cholesterol Outcomes of the Included Articles

**Figure 13:**
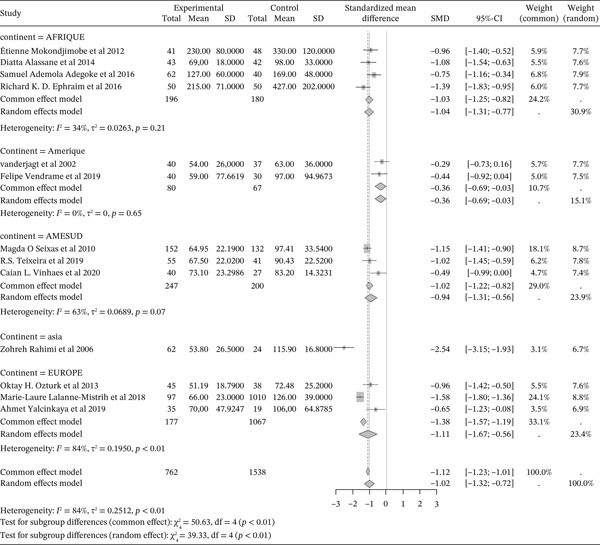
Representation of LDL Cholesterol from included studies. Legend: I^2^ = heterogeneity index; *τ* ^2^ = between‐study variance; SD = Standard; SMD = standardized mean difference; CI 95% = confidence interval (CI) at 95%.

Figure 13 shows the representation of LDL Cholesterol from the included studies. Thirteen studies evaluated the influence of sickle cell disease on plasma LDL cholesterol concentrations, including 4 conducted in Africa, 2 in America, 3 in South America, and 3 in Europe. The total population size of the combined studies is 2,300, including 762 cases and 1,538 controls. The results indicate a significant negative influence of sickle cell disease on plasma LDL cholesterol concentrations, with a reported SMD using a common‐effect model of ‐1.12 [‐1.23; ‐1.01] (p <0.01). The study reporting the strongest influence is that of Rahimi et al. (2006) from Asia, with an SMD of ‐2.54 [‐3.15; ‐1.93] [52]. Conversely, the study showing the weakest influence is that of Vanderjagt et al. (2002) from the USA, with an SMD of ‐0.29 [‐0.73; 0.16] [35]. Significant heterogeneity was observed across the included studies (I^2^>50%).

#### 3.5.4. Sickle Cell Disease and VLDL Cholesterol Outcomes of the Included Articles

**Figure 14:**
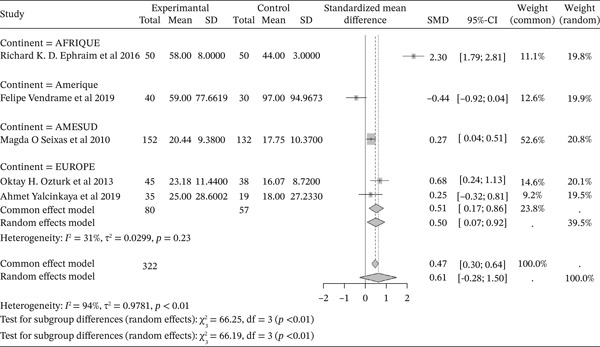
Representation of VLDL Cholesterol from included studies. Legend: I^2^ = heterogeneity index; *τ* ^2^ = between‐study variance; SD = Standard; SMD = standardized mean difference; CI 95% = confidence interval (CI) at 95%.

Figure 14 shows the representation of VLDL Cholesterol from the included studies. Five of the included studies evaluated the influence of sickle cell disease on plasma VLDL Cholesterol concentrations, including 1 from Africa, 1 from America, 1 from South America, and 2 from Europe. The total population size of the combined studies is 591, including 322 cases and 269 controls. The included studies report a moderate influence of sickle cell disease on plasma VLDL Cholesterol concentrations, with a reported SMD using a common‐effect model of 0.47 [0.30; 0.64] (p <0.01). The study reporting the strongest influence is that of Ephraim et al. (2016) from Africa [24], with an SMD of 2.30 [1.79; 2.81]. Conversely, the study reporting the weakest influence is that of Vendrame et al. (2019) from America [34], with an SMD of ‐0.44 [‐0.92; 0.04]. Significant heterogeneity was observed across the included studies (I^2^>50%).

#### 3.5.5. Sickle Cell Disease and Triglycerides Outcomes of the Included Articles

**Figure 15:**
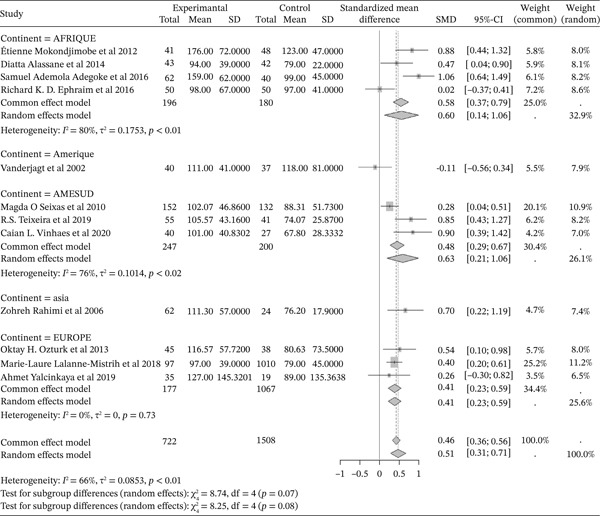
Representation of Triglycerides from included studies. Legend: I^2^ = heterogeneity index; *τ* ^2^ = between‐study variance; SD = Standard; SMD = standardized mean difference; CI 95% = confidence interval (CI) at 95%.

Figure 15 shows the representation of triglycerides from the included studies. Twelve studies evaluated the influence of sickle cell disease on plasma triglyceride concentrations, including 4 conducted in Africa, 1 in America, 3 in South America, 1 in Asia, and 3 in Europe. The total study population for the combined studies is 2,230, including 722 cases and 1,508 controls. The analyses report a moderate influence of sickle cell disease on plasma triglyceride concentrations, with a reported SMD using a common‐effect model of 0.46 [0.36; 0.56] (p <0.01). The study reporting the strongest influence is that of Adegoke et al. (2016) [43] from Africa, with an SMD of 1.06 [0.64; 1.49]. The study reporting the weakest influence is that of Vanderjagt et al. (2002) [35] from the USA, with an SMD of ‐0.11 [‐0.56; 0.34]. Significant heterogeneity was observed across the included studies (I^2^>50%).

#### 3.5.6. Sickle Cell Disease and Triglycerides/HdLc Ratio Outcomes of the Included Articles

**Figure 16:**
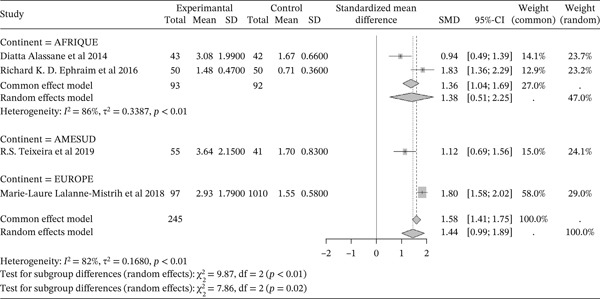
representation of ratio Triglycerides/HdL C from included studies. Legend: I^2^ = heterogeneity index; *τ* ^2^ = between‐study variance; SD = Standard; SMD = standardized mean difference; CI 95% = confidence *interval (CI) at 95%.*

Figure 16 shows the representation of the Triglycerides/HDLc ratio from the included studies. Four studies evaluated the influence of sickle cell disease on the Triglycerides/HDLc ratio, including 2 conducted in Africa, 1 in South America, and 1 in Europe. The total study population for the combined studies is 1,388, including 245 cases and 1,143 controls. The analyses report a significant influence of sickle cell disease on the Triglycerides/HDLc ratio, with a reported SMD using a common‐effect model of 1.58 [1.41; 1.75] (p <0.01). The study reporting the greatest influence is that of Ephraim et al. (2016) from Africa [24], with an SMD of 1.83 [1.36; 2.29]. The study reporting the lowest influence is that of Diatta et al. (2014) [47] from Africa, with an SMD of 0.94 [0.49; 1.39]. Significant heterogeneity was observed across the included studies (I^2^>50%).

## 4. Discussion

Hemolysis in sickle cell patients, resulting from HbS polymerization, is a primary pathophysiological event in sickle cell anemia. This process is associated with significant oxidative stress and inflammation [53]. SCD patients exhibit an imbalance between oxidant production and antioxidant capacity, which is a critical factor in endothelial cell dysfunction, inflammation, vaso‐occlusion, and organ pathology [51, 54–56]. There are multiple potential sources of oxidants in SCD, including accelerated hemoglobin S (HbS) autoxidation [57]; heme/iron‐catalyzed Fenton reactions [58, 59]; and increased expression and activity of various isoforms of Nicotinamide Adenine Dinucleotide Phosphate (NADPH) Oxidase (NOX), Xanthine Oxidase, cytochrome P450, cyclo‐oxygenase, mitochondria, and uncoupled NOS [18, 42, 57, 60–62]. To counteract oxidative stress, erythrocytes possess a self‐sustaining antioxidant defense system, including SOD, CAT, GPx, and GR, along with low‐molecular‐weight antioxidants such as GSH and vitamins [4]. However, given the extent of oxidative stress, the antioxidant system may be impaired, predisposing patients to an increased risk of lipid metabolism dysfunction [63, 64]. Indeed, lipid metabolism in SCD is potentially associated with hemolytic profile and endothelial dysfunction. Similarly, dyslipidemia has been described among patients with different SCD genotypes, with increased low‐density lipoprotein cholesterol (LDL‐C) and decreased high‐density lipoprotein cholesterol (HDL‐C) levels, revealing a potential biomarker of disease severity [37, 65]. This systematic review and meta‐analysis constitutes the first by Cameroonian authors to link published studies on oxidative imbalance in sickle cell patients with lipid profile abnormalities predisposing them to cardiovascular diseases.

The included studies evaluated markers of hemolysis, including LDH, and reported an influence of sickle cell disease on hemolysis compared to normal controls (SMD = 1.15 [0.96; 1.34], p <0.01). This was associated with an elevation of myeloperoxidase (MPO) activity (SMD = 1.03 [0.48; 1.53], p <0.01). Indeed, severe chronic hemolysis is characterized by increased LDH levels in SCD patients and is correlated with elevated MPO concentrations [66–68]. MPO is an enzyme crucial to the oxidative burst of neutrophils during the inflammatory response, and it has been shown to cause oxidative damage to Apo A1 [69].

Furthermore, exploration of the included studies reports a significant elevation of membrane lipid peroxidation products measured as MDA (SMD = 1.13 [0.96; 1.29], p <0.01), with Okorie et al. (2018) from Africa [44] reporting a higher SMD (SMD = 11.99 [9.54; 14.45], p <0.01). Several authors have reported significant elevations of MDA during sickle cell disease compared to normal controls [42, 49, 59, 70, 71]. MDA is considered as a key marker for evaluating the extent of oxidative damage at the cellular level. Accumulation of malondialdehyde disturbs the organization of phospholipids in the human erythrocyte membrane bilayer. Endogenous proteins are targets for free radical attacks via the oxidation of cysteine, methionine, and/or tyrosine residues, forming carbonyls as oxidation products and leading to protein damage. As such, there is a direct link between the generation of free radicals, possibly from sickle RBCs, and increased levels of protein carbonyls [72–74].

Sickle cell pathophysiology affects the oxidant/antioxidant status, with a significant reduction in antioxidant production. This study reported a decrease in the activity of antioxidant enzymes evaluated, including GPx (SMD = ‐1.97 [‐2.32; ‐1.63], p <0.01), GSH (SMD = ‐3.01 [‐3.50; ‐2.52], p <0.01), catalase (SMD = ‐1.39 [‐1.58; ‐1.20], p <0.01), SOD (SMD = ‐1.99 [‐1.92; ‐1.47], p <0.01), and TAC (SMD = ‐1.58 [‐1.90; ‐1.25], p <0.01). This observation of significantly lower antioxidant enzyme levels in sickle cell patients compared to normal controls has been made by several authors [41, 50, 51]. SOD is one of the most effective intracellular enzymatic antioxidants, catalyzing the conversion of superoxide anions to oxygen and H2O2 [4, 58, 75]. The lower levels of erythrocyte SOD and catalase result from the severity of oxidative stress in SCD subjects [50]. As for GSH, a cofactor for GPx in reducing H2O2, it is easily oxidized to glutathione disulfide (GSSG) by oxidants compounds. GPx concentration is also reduced in SCD, with a direct relationship to disease severity. Hemoglobin S, present in the erythrocytes of SCD patients, auto‐oxidizes faster than normal hemoglobin and can result in the generation of more ROS, leading to increased lipid peroxidation and potentially the consumption or inactivation of antioxidant enzymes [56, 76].

In addition, the included studies reported significantly low concentrations of markers for total cholesterol, HDL cholesterol, and LDL, with respective SMDs of ‐1.32 [‐1.42; ‐1.21] (p <0.01), ‐0.84 [‐0.94; ‐0.73] (p <0.01), and ‐1.12 [‐1.23; ‐1.01] (p <0.01), reflecting the negative effect of sickle cell disease on these lipid markers [47, 68, 77, 78]. Conversely, there was a significant increase in plasma triglyceride concentrations and the Triglyceride/HDLc ratio in sickle cell patients compared to controls, with significant SMDs of 0.46 [0.36; 0.56] (p <0.01) and 1.58 [1.41; 1.75] (p <0.01), respectively. This finding has also been reported in previous studies [37, 46]. The sharp decrease in total cholesterol (TC) and LDL‐C usually reported in sickle cell patients could, on one hand, be a consequence of anemia and increased plasma volume due to reduced packed red blood cell volume, and on the other hand, be due to oxidative stress generated by sickle cell disease. Oxidative stress leads to the peroxidation of membrane lipids, which negatively affects plasma lipids and lipoproteins [47, 63, 77, 79]. Thus, hypocholesterolemia may result from the excessive utilization of plasma cholesterol to rebuild red blood cell membranes damaged by lipoperoxidation. Indeed, the increased production of free radicals and lipid peroxidation contributes to the reduction in the half‐life of red blood cells in sickle cell patients [34, 39, 40, 52, 77]. Furthermore, cholesterol biosynthesis is influenced by down‐regulation mechanisms intrinsic to sickle cell disease. The LDL‐C/LDL receptor pathway, which promotes the expression of hydroxymethyl‐glutaryl‐CoA reductase during cholesterol biosynthesis, is regulated to maintain efficiency [22]. The decrease in total cholesterol could also be linked to the activity of lecithin cholesterol acyltransferase (LCAT), an enzyme responsible for membrane cholesterol esterification. LCAT catalyzes the transfer of fatty acids from lecithin to the 3‐hydroxyl group of cholesterol [39, 40]. This acylation reaction allows cholesterol to be transferred from membranes to circulating VLDL as cholesterol esters [63, 77]. Low HDL‐C levels may be due to decreased oxidized lipids and increased reverse cholesterol transport [63, 80, 81]. HDL‐C decline is considered an atherogenic risk factor [27, 82, 83].

Furthermore, the increase in serum triglycerides may be due to decreased lipoprotein lipase activity, which is linked to oxidative stress [84]. Another explanation could be increased production of endogenous lipids, including VLDL and cholesterol; cholesterol would be used to rebuild damaged membranes, while unused triglycerides accumulate [37, 68, 85]. Hypertriglyceridemia may have atherogenic and thrombogenic consequences. Elevated TG/HDL‐C ratios reinforce the pathophysiological interrelationships between hemolysis, inflammation, and lipoproteins in sickle cell anemia (SCA), and highlight the potential role of the lipid profile, particularly the TG/HDL‐C ratio, as a marker of vascular events in SCD patients [9, 38, 40, 77].

All of these results clearly demonstrate that, among the different aspects of pathophysiology, the production of free radicals and lipid peroxidation are major events contributing to the reduction in the half‐life of red blood cells and the appearance of anemia.

## 5. Limitations and Strengths of the Study

Different studies included participants of different age groups and perhaps lifestyles, which could have affected the test results. With all the heterogeneity (*I^2^)* ±37,5%, there was substantial variation in the reported articles’ results. Nonetheless, To our knowledge, this is the first meta‐analysis from Sub‐Saharan Africa to synthesize evidence on oxidative stress and dyslipidemia in SCD, addressing gaps highlighted in prior regional reviews on SCD in Africa and the world.

## 6. Conclusion

Sickle cell disease is a genetic disorder whose intrinsic factors subject patients to a vicious cycle characterized by inflammation, coagulation, fibrinolysis, and abnormalities in the natural anticoagulant system. The objective of this study was to review the literature and perform a meta‐analysis to evaluate oxidative stress, lipid profile, and cardiovascular risk in sickle cell patients. The systematic review of databases and search engines covered a period of 24 years, following the guidelines of PRISMA and the Cochrane Handbook. A total of 25 studies were included, with cases consisting of sickle cell patients and controls consisting of non‐sickle cell individuals. The analyses from these studies reported a substantial influence of sickle cell disease on the oxidant‐antioxidant system, favoring oxidants, as well as an imbalance in lipid homeostasis leading to dyslipidemia and a predisposition to atherogenic risk when compared to normal controls, with SMDs ranging from moderate to significant. These results provide further insight into oxidative stress and lipid profile abnormalities in sickle cell disease and can serve as a foundation for informed decision‐making to improve patient care. Moreover, these findings could guide future research to better understand the mechanisms linking the oxidant‐antioxidant system, dyslipidemias, and atherogenic risk, thereby helping clinicians optimize the management of patients with sickle cell disease.

## Author Contributions

Romaric De Manfouo Tuono, Josué Louokdom Simo, and Maryline Seuko Njopwouo contributed to the study design, article selection, data extraction, statistical analysis, data interpretation, and manuscript writing. Claude Tagny Tayou supervised the study and reviewed the manuscript. All authors read and approved the final version of the manuscript. Romaric De Manfouo Tuono had full access to all the data in this study and took complete responsibility for the integrity of the data and the accuracy of the data analysis.

## Funding

No funding was received for this manuscript.

## Disclosure

Transparency Statement. Romaric Tuono De Manfouo affirms that this manuscript is an honest, accurate, and transparent account of the study being reported; that no important aspects of the study have been omitted; and that any discrepancies from the study as planned (and, if relevant, registered) have been explained”.

## Ethics Statement

Not applicable.

## Consent

Not applicable.

## Conflicts of Interest

The authors declare that they have no conflicts of interest regarding the publication of this manuscript.

## Data Availability

The data that support the findings of this study are available from the corresponding author upon reasonable request.
